# Evaluation of serum nucleoside diphosphate kinase A for the detection of colorectal cancer

**DOI:** 10.1038/srep26703

**Published:** 2016-05-25

**Authors:** Olalla Otero-Estévez, Loretta De Chiara, Leticia Barcia-Castro, María Páez de la Cadena, Francisco Javier Rodríguez-Berrocal, Joaquín Cubiella, Vicent Hernández, Vicenta Soledad Martínez-Zorzano

**Affiliations:** 1 Department of Biochemistry, Genetics and Immunology, Universidade de Vigo, Vigo, Spain; 2 Department of Gastroenterology; Complexo Hospitalario Universitario de Ourense. Instituto de Investigación Biomédica Ourense-Pontevedra-Vigo, Ourense, Spain; 3 Department of Gastroenterology; Xerencia de Xestión Integrada de Vigo. Instituto de Investigación Biomédica Ourense-Pontevedra-Vigo, Vigo, Spain

## Abstract

We previously described the over-expression of nucleoside diphosphate kinase A (NDKA) in tumours and serum from colorectal cancer (CRC) patients, suggesting its use as biomarker. In this study we evaluated the diagnostic accuracy of serum NDKA to detect advanced neoplasia (CRC or advanced adenomas). Furthermore, the performance of NDKA was compared with the faecal immunochemical test (FIT). The study population included a case-control cohort and a screening cohort (511 asymptomatic first-degree relatives of CRC patients that underwent a colonoscopy and a FIT). Serum NDKA was elevated in CRC patients in the case-control cohort (*p* = 0.002). In the screening cohort, NDKA levels were higher for advanced adenomas (*p* = 0.010) and advanced neoplasia (*p* = 0.006) compared to no neoplasia. Moreover, elevated NDKA was associated with severe characteristics of adenomas (≥3 lesions, size ≥ 1 cm or villous component). Setting specificity to 85%, NDKA showed a sensitivity of 30.19% and 29.82% for advanced adenomas and advanced neoplasia, respectively. NDKA combined with FIT (100 ng/mL cut-off) detected advanced adenomas and advanced neoplasia with 45.28% and 49.12% sensitivity, with specificity close to 90%. The combination of serum NDKA and FIT can improve the detection of advanced neoplasia, mainly for lesions located on the proximal colon, in asymptomatic individuals with CRC family-risk.

Colorectal cancer (CRC) is the third most common cancer in men and the second in women, with annually more than 1.3 million new cases and near 700,000 deaths worldwide^1^. One of the most important prognostic factors of CRC is the stage at diagnosis, with a 5-year relative survival rate greater than 90% for patients diagnosed at early stages^2^. In addition, the detection and removal of premalignant advanced adenomas (AA) has shown to reduce the incidence of CRC^3^. Therefore, screening programs are particularly important for this pathology. Colonoscopy is considered the most accurate test for the early detection of both cancer and clinically significant adenomas^4^. However, the adherence to this procedure is very low^5^, even for family-risk populations who have an increased risk of developing this neoplasia^6^. Nowadays, the most used non-invasive screening strategy is the faecal immunochemical test (FIT), which is highly sensitive for detecting CRC but inadequate for AA^7^,^8^,^9^. Additionally, it has been reported that the faecal test performs better detecting distal lesions compared with proximal ones^10^,^11^. Therefore, the improvement of non-invasive screening tools is necessary to increase the detection of these precursor lesions. In particular, the development of blood-based tests with an optimal diagnostic performance would enhance the adherence to CRC screening programs and consequently reduce the incidence and mortality of CRC^12^.

The NME/NM23 nucleoside diphosphate kinase 1 gene (*NME1*) is located on chromosome 17q21.3 and was the first metastasis suppressor gene described^13^. This gene encodes a protein of 18.5 kDa that is called nucleoside diphosphate kinase A (NDKA; EC: 2.7.4.6) because of its catalytic activity^14^. Moreover, the NDKA has other enzymatic activities like histidine protein kinase^15^ and 3′–5′ exonuclease^16^, both relevant for its anti-metastatic function^17^. Among other functions, this molecule is also involved in cell proliferation and differentiation^14^,^17^. Low expression of both NDKA protein and mRNA has been associated with high metastatic potential and poor prognosis in different tumours, such as ovarian cancer, melanoma, hepatocellular, breast and gastric carcinomas^17^,^18^,^19^. In contrast, highly expressed NDKA is related to a more aggressive disease in neuroblastoma and haematological malignancies [reviewed in 20].

NDKA is also detected in blood, though the mechanism responsible for its secretion into the extracellular space is not clear^17^,^20^. High levels of serum NDKA have been reported in individuals with haematological cancers like leukaemia and lymphoma compared with healthy controls, proposing this molecule as a prognostic factor^20^,^21^. Moreover, NDKA was suggested as a marker for early detection of kidney cancer due to its elevation in the plasma of patients^22^.

In the case of CRC, several studies have reported significantly higher NDKA protein and/or mRNA expression in tumour tissue in comparison to normal mucosa^23^,^24^,^25^,^26^,^27^,^28^. In a previous work, our group identified through a proteomic study the NDKA, observing increased protein levels in tumour tissue compared to healthy mucosa. We also analysed the relation between NDKA and the tumorigenic process using the Caco-2 cell line differentiation model, reporting an over-expression of the protein in extracts of undifferentiated tumour-like cells as well as in its secretome. In addition, we detected by Western blot an increased amount of NDKA in serum of CRC patients, suggesting the potential use of this molecule as a CRC serum marker^29^.

Therefore, in this study we measured serum NDKA using a sensitive method such as ELISA in a case -control cohort, and in an asymptomatic screening population with family history of CRC that included diverse colorectal pathologies, to evaluate the diagnostic capability of this biomarker to detect advanced neoplasia (AN: CRC or AA). Furthermore, NDKA was compared to FIT in terms of diagnostic performance.

## Results

### Serum NDKA levels in the case-control cohort

**Figure 1:**
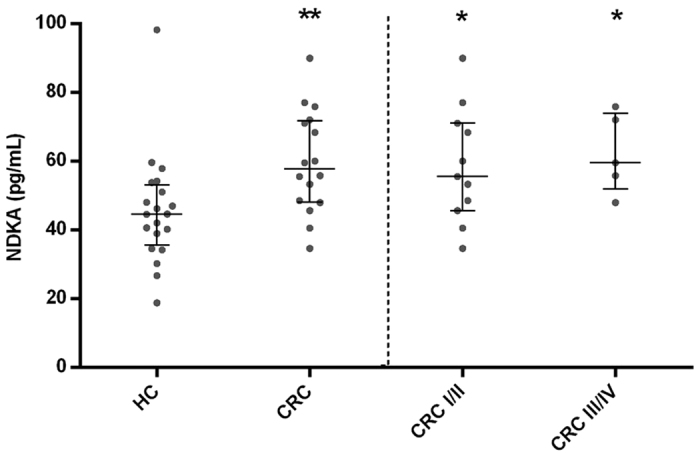
Scatterplot representation of serum NDKA levels in the case-control cohort. Lines represent median values and whiskers indicate the interquartile range. HC: healthy controls; CRC: colorectal cancer; I/II: stage I and II; III/IV: stage III and IV; *p < 0.05 and **p < 0.01 for comparisons of NDKA levels with healthy controls (Mann-Whitney U test).

The serum NDKA levels were found elevated in CRC patients (median 57.76 pg/mL; IQ range 48.19–71.84 pg/mL) compared with healthy controls (median 44.69 pg/mL; IQ range 35.74–53.18 pg/mL), resulting in statistically significant differences (Mann–Whitney U-test, *p* = 0.002; Fig. 1). Significant differences were also observed comparing stage I/II (median 55.66; IQ range 45.74–71.10 pg/mL) and stage III/IV patients (median 59.65 pg/mL; IQ range 51.95–74.04 pg/mL) with the healthy control group (Mann–Whitney U-test, *p* = 0.025 and *p* = 0.012, respectively; Fig. 1). The diagnostic performance of serum NDKA for the detection of CRC in this case-control cohort resulted in an AUC of 0.731 (95% CI: 0.580–0.851). In relation to tumour characteristics including tumour stage, invasion of the tumour (pT), lymph node affectation (pN), distant metastasis (pM), differentiation grade or location, no differences were found in the serum NDKA concentration (Supplementary Table S1).

### Serum NDKA levels in the screening cohort

The serum NDKA concentration was analysed in the family-risk screening cohort regarding age, gender, and familial risk of the individuals (Table 1). Slightly increased NDKA levels were observed with advanced ages, though no statistical significant differences were found (Kruskal-Wallis test, *p* = 0.259). Regarding gender, no differences were either detected in the NDKA serum levels (Mann–Whitney U-test, *p* = 0.354). In relation to the age and the number of first-degree relatives with CRC, there were no statistical significant differences in the NDKA concentration (Kruskal-Wallis test, *p* = 0.985).

Table 2 summarises the median and IQ range of serum NDKA levels according to the colonoscopy findings. The no neoplasia group that included individuals with no colorectal findings (median 57.37 pg/mL) and benign pathologies (median 56.19 pg/mL) showed low serum NDKA concentration (median 56.90 pg/mL). The conditions grouped within the benign pathologies category showed no differences compared to the group with no colorectal findings (Mann–Whitney U-tests, *p* > 0.05).

A continuous increase in the serum NDKA levels was observed from individuals with no neoplasia, followed by non-advanced adenomas (NAA; median 58.34 pg/mL), AA (median 66.93 pg/mL), and finally CRC (median 66.98 pg/mL). However, there were no statistical significant differences between the small group of individuals with CRC and the group with no neoplasia included in the screening cohort (Mann–Whitney U-test, *p* = 0.262). In contrast, AA showed a statistical significant increase compared with the no neoplasia group (Mann–Whitney U-test, *p* = 0.010). Likewise, AN (median 66.93 pg/mL) also resulted in statistical significant differences compared with no neoplasia (Mann–Whitney U-test, *p* = 0.006).

### Relationship between serum NDKA and the characteristics of adenomas in the screening cohort

Based on the increase observed in the levels of serum NDKA from non-advanced to advanced adenomas (Mann–Whitney U-test, *p* = 0.072), the concentration of this molecule was also analysed according to the characteristics of the lesions. As shown on Table 3, the highest median NDKA values were observed for the more severe characteristics of adenomas. Individuals with 3 or more adenomas had higher NDKA levels compared to individuals with 1–2 adenomas, though the difference was not significant (Mann–Whitney U-test, *p* = 0.212). Regarding size, a significant increase of serum NDKA was observed in adenomas larger than 1 cm in relation to smaller adenomas (median 68.32 pg/mL *vs* 58.32 pg/mL; Mann–Whitney U-test, *p* = 0.035). Patients bearing adenomas with villous component showed elevated median NDKA levels (66.93 pg/mL) in contrast to those with tubular histology (59.04 pg/mL), resulting in a difference near statistical significance (Mann–Whitney U-test, *p* = 0.065). Finally, no differences in serum NDKA were found between distal and only proximal location of the lesions (Mann–Whitney U-test, *p*=0.648).

### Diagnostic performance of serum NDKA based on the screening cohort

The diagnostic performance of serum NDKA for the detection of AN and AA was analysed in the screening population with family history of CRC. The ROC curves for AN and AA resulted in an AUC of 0.608 (95% CI: 0.564–0.650) and 0.604 (95% CI: 0.560–0.647), respectively. Table 4 shows the performance of serum NDKA for the different cut-off points studied resulting from setting specificity close to 85%, 90%, 95% and also for the cut-off point based on the Youden index. At the ≥76.07 pg/mL cut-off, 29.82% of AN and 30.19% of AA were detected with a specificity of 85.02%. Fixing specificity to 95.15% (NDKA ≥ 94.79 pg/mL), we identified 17.54% of AN and 16.98% of AA. The highest sensitivity was obtained for the Youden index cut-off (NDKA ≥ 62.59 pg/mL), resulting in 63.16% for AN and 62.26% for AA, though the corresponding specificity (65.20%) is very low for a screening program. Moreover, for all the cut-off points studied the negative predictive values were greater than 90%.

### Diagnostic performance of FIT based on the screening cohort

The diagnostic performance of FIT was also studied in the family-risk screening cohort (Table 5). Based on the established 100 ng haemoglobin/mL cut-off (20 μg haemoglobin/g faeces), 36.84% of the AN cases were detected, with a 98.24% specificity. For AA detection this cut-off resulted in a sensitivity of 32.08% (95% CI: 19.9–46.3), with the same 98.24% specificity. FIT detected 41.03% of distal AA in contrast with the limited 7.14% of proximal AA.

### Diagnostic performance of the combination of serum NDKA and FIT based on the screening cohort

The diagnostic parameters for the combined markers are shown on Table 5 and were analysed for both the 82.21 pg/mL and the 94.79 pg/mL NDKA cut-offs. Sensitivity values increased to 45.61% and 41.51% for detecting AN and AA for the combination of NDKA (94.79 pg/mL cut-off) and FIT compared to only FIT, resulting almost statistically significant (McNemar test, *p* = 0.0625 in both cases). These differences resulted significant when the 82.21 pg/mL NDKA cut-off was used in the combination (McNemar test, *p* = 0.0156 for both AN and AA), reaching a 49.12% and 45.28% sensitivity, respectively. The combined use of NDKA and FIT considerably enhanced the detection of proximal AA up to 28.57% for either cut-offs, and slightly improved the sensitivity for distal AA reaching 46.15% and 51.28% for the 94.79 pg/mL and 82.21 pg/mL NDKA cut-off, respectively.

## Discussion

Since the NDKA codifying gene (*NME1*) was first described as a putative tumour suppressor implicated in the metastatic process in melanoma and breast carcinoma^13^, the concern about the role of this protein in these and other human cancers has extended. In CRC many studies managing small series of patients have centred on the tissue expression of NDKA as a prognostic factor for metastasis, with inconclusive results^25^,^28^,^30^,^31^,^32^,^33^,^34^.

Our interest for this molecule derives from a proteomic study from our group that searched for CRC biomarkers using paired tumour-mucosa tissues. Among the soluble proteins identified was NDKA, which was increased in tumour compared to healthy mucosa, and was as well elevated in undifferentiated cells in relation to differentiated ones in the Caco-2 cell line differentiation model. The fact that NDKA also appeared over-expressed in the secretome of these undifferentiated cells, besides its detection in serum samples from CRC patients, added new evidence for its potential utility in the diagnosis of CRC^29^. Hence, our study is the first that analyses the serum levels of NDKA in a wide variety of colorectal pathologies including CRC and adenomas with diverse clinicopathological characteristics, and also evaluates the diagnostic performance of the molecule for the detection of AN in asymptomatic subjects with a family history of CRC. In addition, since the individuals included in our screening cohort participated in a study evaluating FIT as a diagnostic test^7^, the outcome of our potential marker NDKA could be compared with that of the faecal test.

Once confirmed that the commercial ELISA kit was suitable to quantify NDKA in our serum samples, we first compared the levels of this molecule in a small group of CRC patients and healthy controls. Cancer patients exhibited higher serum NDKA levels as expected, in line with the findings reported by our group and others for the protein in tumour tissue compared to normal mucosa using Western blot or immunohistochemistry techniques^26^,^28^,^29^,^31^, as well as for mRNA by Northern blot and RT-PCR^24^,^25^,^27^. Regarding the characteristics of the tumour, we did not find any association between serum NDKA and tumour stage, TNM classification, differentiation grade or location, consistent with that reported by others analysing NDKA expression in tumour tissue^23^,^28^,^34^,^35^,^36^.

In relation to our interest in NDKA for discriminating CRC patients, we found significant differences in the serum levels comparing stage I/II with healthy controls, though the number of subjects is very modest. The potential utility of this protein for the early diagnosis of CRC was also evidenced, with an AUC greater than 0.7.

In view of this promising but limited result due to the reduced number of cases, NDKA serum levels were further evaluated in an asymptomatic family-risk screening cohort including 511 individuals. First of all, no differences were observed regarding age or gender, as stated by other authors for NDKA from tumour specimens^30^,^31^,^33^. Additionally, the familial risk categories showed no association with NDKA. In relation to the NDKA serum levels according to the colonoscopy result, individuals with no neoplasia (including no colorectal findings and benign pathologies) showed similar concentrations among them. This characteristic is of great value for diagnosis since one of the most common handicaps of biomarkers is their alteration in the presence of benign conditions (false positive results)^37^,^38^. Besides this advantage, we also found that NDKA levels increased progressively from no neoplasia – non-advanced adenomas – advanced adenomas – cancer, in correspondence to the normal epithelium – adenoma – carcinoma sequence. A continuous increase in the expression of NDKA from mucosa – adenoma – CRC was also previously reported for tissue samples based on immunohistochemistry^31^ and cDNA arrays^39^, suggesting that NDKA may play an important role in this sequential progression.

In our study a more detailed analysis was conducted in relation to the clinicopathological characteristics of adenomas, which was not previously performed by others. We analysed serum NDKA levels in a considerable total of 173 adenomas, observing a trend towards increased median concentrations associated with the characteristics that define AA and high-risk adenomas. This tendency of higher NDKA levels is in line with the adenoma-carcinoma sequence and supports its implication in the neoplastic transformation, providing new evidence for the utility of this molecule in the diagnosis of AA, besides CRC. Importantly, differences were not found for distal and proximal lesions, resulting of great importance given that the dependence on location is a limitation of FIT^10^,^11^.

Considering the ability of serum NDKA to discriminate individuals with no neoplasia from those with AN, we evaluated its diagnostic performance in the family-risk screening cohort. ROC curves for the diagnosis of AN and AA rendered AUC values close to 0.61, reaching sensitivities for AN detection under 30% for 85, 90 or 95% specificities.

Taking advantage of our study design for the screening cohort, we were able to compare the performance of NDKA with that of FIT for the detection of AN and AA. We used the established 100 ng haemoglobin/mL cut-off (20 μg haemoglobin/g faeces)^40^, that reached a sensitivity for detecting AN slightly higher than that seen for NDKA, and resulting in a superior specificity. However, FIT showed limited capability to detect proximal AA since only 7.14% of these lesions were identified.

Though individually serum NDKA did not show the optimal performance expected for a screening test, the combination of this new marker with FIT resulted in a sensitivity superior to 45% for both NDKA cut-offs analysed for the detection of AN. Regarding specificity, though this parameter was affected when markers were combined, a value close to 90.0–95.0% which is typically recommended in a screening setting was obtained. Based on our asymptomatic screening cohort, the combination of serum NDKA and FIT allowed the identification of the 4 CRC cases besides 41.51% (22/53) and 45.28% (24/53) of the AA (94.79 ng/mL or 82.21 pg/mL NDKA cut-off, respectively), with a considerable increase in the detection of proximal adenomas compared with only FIT.

In the last years many studies have evaluated new potential markers with the aim of finding alternative CRC screening tools that could overcome the disadvantages of actual methods of colonoscopy or FIT. To date, the most promising markers are the methylated SEPT9 DNA measured in plasma^41^ and the multitarget stool DNA test^42^. Though sensitivities for CRC close to or higher than 50% were reported in these studies, the performance for detecting AA was only 11% for the blood-based test (specificity 91.5%)^41^ and 42% for the stool test (specificity 86.6%)^42^. In relation to other candidate serum markers, protein panels reached 42% sensitivity for CRC^43^,^44^ and 9% for AA^43^, setting specificity to 95%. More recently, serum autoantibody panels reported 10% sensitivity for AN^45^ and 48% sensitivity for CRC^46^, both at 90% specificity.

Despite some candidates seem more useful than others, several reviews^47^,^48^,^49^,^50^ agree about the inexistence of an optimal biomarker with a suitable sensitivity and specificity, and state that studies may have reported imprecise performance characteristics due to: small sample sizes, case-control cohorts typically symptomatic that do not include other colorectal pathologies, healthy controls with no colonoscopy, markers evaluated in only one study, adenomas included in very few studies, and finally, no large-scale multi-centric validation performed to confirm results.

Our study design does not have the above limitations, on the contrary, as suggested recently by Shah *et al.*^47^ for studies evaluating biomarkers, we measured serum NDKA in a screening population linked to a national screening program and were able to perform a direct comparison of our experimental marker with the up-to-date established FIT. Hence, our results on the performance of NDKA are based on a considerable family-risk cohort examined through colonoscopy, which included a wide variety of pathologies besides a significant number of AA cases, constituting the strong point of our work.

On the other hand, one of the limitations of our study is the reduced number of individuals included in the case-control cohort. However, the measurement of serum NDKA levels in this group was conceived to verify the correct quantification of the molecule using the commercial ELISA assay, and to corroborate the difference in serum NDKA levels between healthy controls and CRC patients evidenced in our previous work by Western blot^29^. In the screening cohort, a small number of CRC patients were also found, corresponding to a prevalence of 0.78% which is expected in a familial-risk screening context (0.65–0.7%)^51^,^52^. Nevertheless, since the detection of AN is the goal of this type of approach, the total of 57 cases of CRC together with AA seem suitable for the analysis.

In conclusion, we report a complete evaluation of serum NDKA for CRC early detection, not previously reported. Though NDKA by itself did not show the optimal performance expected for a screening test, the combination of this new marker with FIT can contribute to improve the detection of AN in an asymptomatic family-risk cohort, mainly for the proximal lesions. Our findings should be confirmed in a larger multi-centric cohort.

## Methods

### Study population

The study population included a case-control cohort and a screening cohort, recruited from both *Complexo Hospitalario Universitario de Ourense* and *Xerencia de Xestión Integrada de Vigo* hospitals. The first comprised 16 patients with CRC (11 men, aged 52–84 years, median 66.0 years) and 20 healthy control individuals (12 men, aged 52–67 years, median 58.3 years). CRC patients included symptomatic individuals classified according to the AJCC staging system^53^ as: 2 stage I, 9 stage II, 1 stage III and 4 stage IV. Three tumours had a proximal location (proximal to the splenic flexure of the colon) while 13 tumours were located in the distal colon. Healthy controls corresponded to individuals with no colorectal findings, verified by colonoscopy.

On the other hand, the screening population consisted of a prospective, controlled, double-blinded cohort that included 511 asymptomatic individuals with at least one first-degree relative with CRC recruited from the hospital of *Complexo Hospitalario Universitario de Ourense*, as described in Castro *et al.*^7^. A stool sample was obtained for each individual one week before colonoscopy. The faecal occult blood test used was the quantitative immunochemical OC-sensor test (Eiken Chemical; Tokyo, Japan). The colonoscopy performed by experienced endoscopists, blind to the FIT, allowed the classification of individuals according to the most advanced lesion as: 4 (0.8%) CRC cases, 53 (10.4%) individuals with AA (adenomas with high grade dysplasia, ≥10 mm and/or with villous histology component), 120 (23.5%) with NAA, 161 (31.5%) with benign pathologies [including 5 (1.0%) inflammatory polyps, 39 (7.6%) hyperplasic polyps, 67 (13.1%) haemorrhoids, 44 (8.6%) diverticula and 6 (1.2%) with other benign pathologies], and 173 (33.9%) subjects with no colorectal findings. According to the tumour stage, there were 2 stage I cases, 1 stage II case and 1 stage III case, all of them located on the distal colon. In relation to AA, these were classified as ‘proximal’ when located only proximal to the splenic flexure (n = 14), and ‘distal’ when found only in distal colon or in both distal and proximal colon (n = 39).

An informed consent was obtained from each individual and anonymity was warranted. The study was conducted in accordance with the Helsinki Declaration and the clinical-ethical practices of the Spanish Government, and was approved by the Galician Ethical Committee for Clinical Research.

### Serum collection and NDKA measurement

A blood sample was collected from all the individuals included in both cohorts. Blood was coagulated at room temperature and then centrifuged at 2,000 *g* during 20 min to obtain the serum, which was stored at −20 °C until used.

The serum NDKA concentration (pg/mL) was measured with Human Nucleoside Diphosphate Kinase A (NME1) ELISA kit (Cusabio Biotech Co., Wuhan, China) according to the manufacturer’s instructions and blinded to the colonoscopy and FIT results. Colorimetric quantification was performed by duplicate and absorbance was measured at 450/570 nm in a microplate reader (model 550; Bio-Rad; Hercules, CA, USA).

### Data analysis

All patient information and measurements were registered in a specific database. The statistical analyses were performed with SPSS software (v.20.0, SPSS Inc., Chicago, USA). Continuous variables were presented as median and IQ range. The non-parametric Mann-Whitney U test was used to compare two groups and the non-parametric Kruskal–Wallis test was performed for multiple group comparisons. The capability of NDKA to separate patients from healthy individuals was assessed with the receiver operating characteristic (ROC) curve using MedCalc (v.14.12.0, Ostend, Belgium). The diagnostic parameters were calculated setting specificity close to 85%, 90% and 95%, and also for the Youden index. In the case of FIT, a cut-off of 100 ng haemoglobin/mL (20 μg haemoglobin/g faeces) was used which is the standard recommended by the manufacturer^40^. The criteria used to combine NDKA and FIT was based on their individual cut-offs: a test was considered positive when at least one of the markers was positive (NDKA and/or FIT), whereas a test was negative when both markers resulted negative. McNemar test was used to compare the sensitivities of the combination of NDKA and FIT in relation to only FIT for the detection of AN or AA. All statistical analyses were two-sided and *p* values ≤ 0.05 were considered statistically significant.

## Additional Information

**How to cite this article**: Otero-Estévez, O. *et al.* Evaluation of serum nucleoside diphosphate kinase A for the detection of colorectal cancer. *Sci. Rep.* **6**, 26703; doi: 10.1038/srep26703 (2016).

## Supplementary Material

Supplementary Table S1

## Figures and Tables

**Table 1:** Serum NDKA levels in the screening cohort according to demographic and clinical characteristics.

Characteristics	N	Median (pg/mL)	IQR (pg/mL)	*P*
Age (years)				
≤49	177	55.88	45.91–67.76	0.259^a^
50–59	172	58.80	46.64–70.32	
≥60	162	60.25	47.86–70.95	
Gender				
Male	210	58.80	47.15–71.14	0.354^b^
Female	301	58.31	46.57–68.36	
Familial risk				
1 FDR ≥ 60 years	340	58.80	46.88–69.72	0.985^a^
1 FDR < 60 years	117	57.23	46.38–69.44	
≥2 FDR	54	56.15	45.02–74.58	

IQR: interquartile range; ^a^Kruskal-Wallis test, ^b^Mann-Whitney U test. FDR: first-degree relative.

**Table 2:** Serum NDKA levels in the screening cohort according to the colorectal findings.

Colorectal findings	N	Median (pg/mL)	IQR (pg/mL)	*P*
**No neoplasia**	334	56.90	46.88–66.94	
No colorectal findings	173	57.37	46.88–69.11	
Benign pathologies	161	56.19	46.86–65.44	0.542^b^
Inflammatory Polyps	5	55.24	49.00–71.27	0.944^b^
Hyperplasic Polyps	39	55.05	42.21–65.00	0.218^b^
Haemorrhoids	67	55.43	47.14–64.51	0.338^b^
Diverticula	44	60.70	47.26–71.76	0.472^b^
Other	6	60.39	46.48–83.07	0.548^b^
**Non-advanced adenomas**	120	58.34	45.12–71.06	0.499^a^
**Advanced neoplasia**	57	66.93	47.54–82.49	0.006^a^
Advanced adenomas	53	66.93	47.54–82.49	0.010^a^
Cancer	4	66.98	49.18–91.81	0.262^a^

IQR: interquartile range; *p* values correspond to Mann-Whitney U test for comparisons with the no neoplasia group (a) or the no colorectal findings group (b).

**Table 3:** Serum NDKA levels in the screening cohort according to the characteristics of adenomas.

Variable	N	Median (pg/mL)	IQR (pg/mL)	*P*
Number				
1–2	141	60.17	45.94–71.36	0.212
3 or more	32	63.17	44.59–90.66	
Size				
<1 cm	126	58.32	44.67–71.70	0.035
≥1 cm	47	68.32	48.09–83.39	
Histology				
Tubular	146	59.04	44.99–73.06	0.065
Villous or tubulovillous	27	66.93	52.09–84.76	
Location				
Distal	132	60.26	45.58–73.45	0.648
Only proximal	41	62.47	44.15–73.83	

IQR: interquartile range; *p* values correspond to Mann-Whitney U test.

**Table 4:** Diagnostic performance of serum NDKA for the detection of advanced neoplasia and advanced adenomas based on the screening cohort.

Cut-off (pg/mL)	Specificity% (95% CI)	Advanced neoplasia			Advanced adenomas		
		Sensitivity % (95% CI)	PPV % (95% CI)	NPV % (95% CI)	Sensitivity % (95% CI)	PPV % (95% CI)	NPV % (95% CI)
≥62.59^a^	65.20 (60.6–69.6)	63.16 (49.3–75.6)	18.63 (13.3–24.8)	93.35 (90.1–95.9)	62.26 (47.9–75.2)	17.35 (12.2–23.4)	93.64 (90.4–96.1)
≥76.07	85.02 (81.4–88.2)	29.82 (18.4–43.4)	20.07 (12.1–30.1)	90.57 (87.4–93.2)	30.19 (18.3–44.3)	19.12 (11.3–29.1)	91.21 (88.1–93.8)
≥82.21	90.09 (87.0–92.7)	24.56 (14.1–37.8)	23.81 (13.6–36.6)	90.45 (87.4–93.0)	24.53 (13.8–38.3)	22.50 (12.5–35.3)	91.05 (88.1–93.6)
≥94.79	95.15 (92.8–96.9)	17.54 (8.7–29.9)	31.32 (16.1–50.0)	90.15 (87.2–92.7)	16.98 (8.1–29.8)	29.12 (14.2–48.0)	90.71 (87.8–93.2)

^a^Younden index; PPV: positive predictive value; NPV: negative predictive value.

**Table 5:** Diagnostic performance of the combination of NDKA and FIT for the detection of advanced neoplasia and advanced adenomas based on the screening cohort.

Cut-off	Specificity % (95% CI)	Advanced neoplasia (n = 57)			Advanced adenomas (n = 53)				
		Sensitivity % (95% CI)	PPV % (95% CI)	NPV % (95% CI)	Sensitivity % (95% CI)	PPV % (95% CI)	NPV % (95% CI)	Proximal n = 14 (% detection)	Distal n = 39 (% detection)
FIT ≥100 ng/mL	98.24 (96.6–99.2)	36.84 (24.4–50.7)	72.44 (44.1–86.0)	92.53 (91.3–94.3)	32.08 (19.9–46.3)	68.02 (46.5–85.1)	92.53 (89.9–94.8)	7.14	41.03
NDKA ≥82.21 pg/mL and/or FIT ≥100 ng/mL	88.32 (84.9–91.1)	49.12 (35.8–62.6)	34.57 (24.6–46.0)	93.26 (90.3–95.4)	45.28 (31.8–59.4)	31.12 (21.4–42.9)	93.26 (90.3–95.4)	28.57	51.28
NDKA ≥94.79 pg/mL and/or FIT ≥100 ng/mL	93.17 (90.3–95.2)	45.61 (32.6–59.2)	45.61 (32.6–59.2)	93.17 (90.3–95.2)	41.51 (28.4–55.8)	41.49 (28.4–55.8)	93.17 (90.3–95.2)	28.57	46.15

PPV: positive predictive value; NPV: negative predictive value.
