# Supplementary material for: Evaluation of serum nucleoside diphosphate kinase A for the detection of colorectal cancer
Source: Sci Rep. 2016 May 25;6:26703. doi: 10.1038/srep26703 (PMC4879623; doi:10.1038/srep26703)
Supplement: Supplementary Table S1 [file srep26703-s1.pdf]

Supplementary information

Title: Evaluation of serum nucleoside diphosphate kinase A for the early detection of colorectal cancer.

Authors: Olalla Otero-Estévez[†], Loretta De Chiara[†], Leticia Barcia-Castro, María Páez de la Cadena, Francisco Javier Rodríguez-Berrocal, Joaquín Cubiella, Vicent Hernández, Vicenta Soledad Martínez-Zorzano*.

Supplementary Table S1. Serum NDKA levels of CRC patients from the case-control cohort according to the characteristics of the tumour.

Variable	N	Median (pg/mL)	IQR (pg/mL)	*P*
**Stage**				
I	2	62.06	55.66-68.45	0.915[a]
II	9	53.38	43.17-74.11	
III	1	59.65		
IV	4	63.98	50.00-75.02	
**pT**				
T1	2	62.06	55.66-68.45	0.916[a]
T2	0			
T3	8	56.52	42.61-72.87	
T4	6	63.48	47.47-73.07	
**pN**				
N0	11	55.66	45.74-71.10	0.406[a]
N1	2	53.85	48.04-59.65	
Nx	3	72.09	55.86-75.99	
**pM**				
M0	12	57.66	46.46-70.44	0.599[b]
M1	4	63.98	50.00-75.02	
**Differentiation Grade**				
Well	1	68.45		0.808[a]
Moderate	14	55.76	47.47-73.07	
Poor	1	60.14		
**Location**				
Distal	13	59.65	47.18-73.55	0.900[b]
Proximal	3	55.66	48.04-72.09	

IQR: interquartile range; [a]Kruskal-Wallis test, [b]Mann-Whitney U test.
